# Supplementary material for: Maintaining ecological stability for sustainable economic yields of multispecies fisheries in complex food webs
Source: Nat Commun. 2025 Sep 25;16:8425. doi: 10.1038/s41467-025-64179-3 (PMC12462472; doi:10.1038/s41467-025-64179-3)
Supplement: Supplementary file 1 — Supplementary Information [file 41467_2025_64179_MOESM1_ESM.pdf]

SI 1 Evenness of the distribution of fish biomass caught across the three fishery fleets depending on the fishery scenario

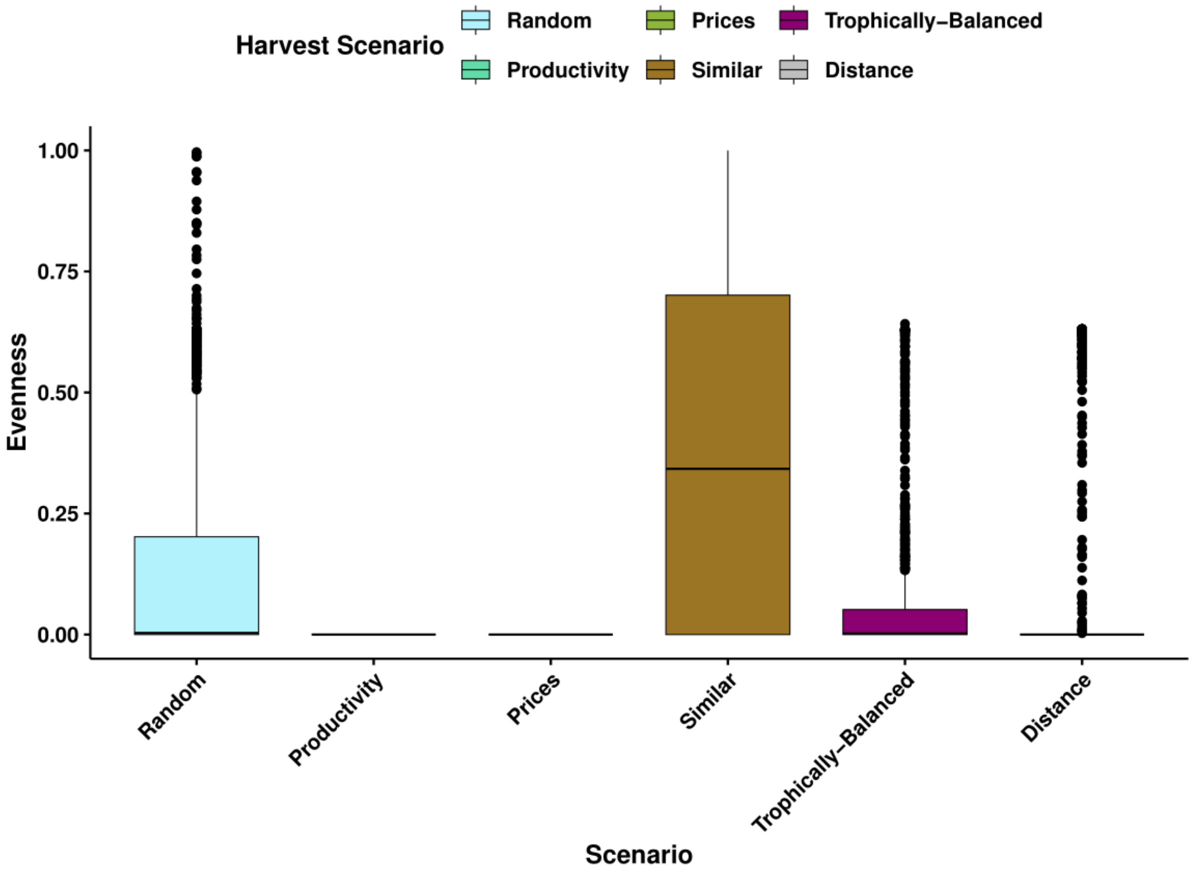

Supplementary Figure S1. Evenness of fish biomass caught by the three fisheries for each scenario (n = 800 food webs for each scenario). Here, and for each bar plots of this document, The median and box limits correspond to the 0.25 and 0.75 quantiles. Whiskers represent the 1.5 interquartile range.

SI 2 Coefficient of variation per scenario

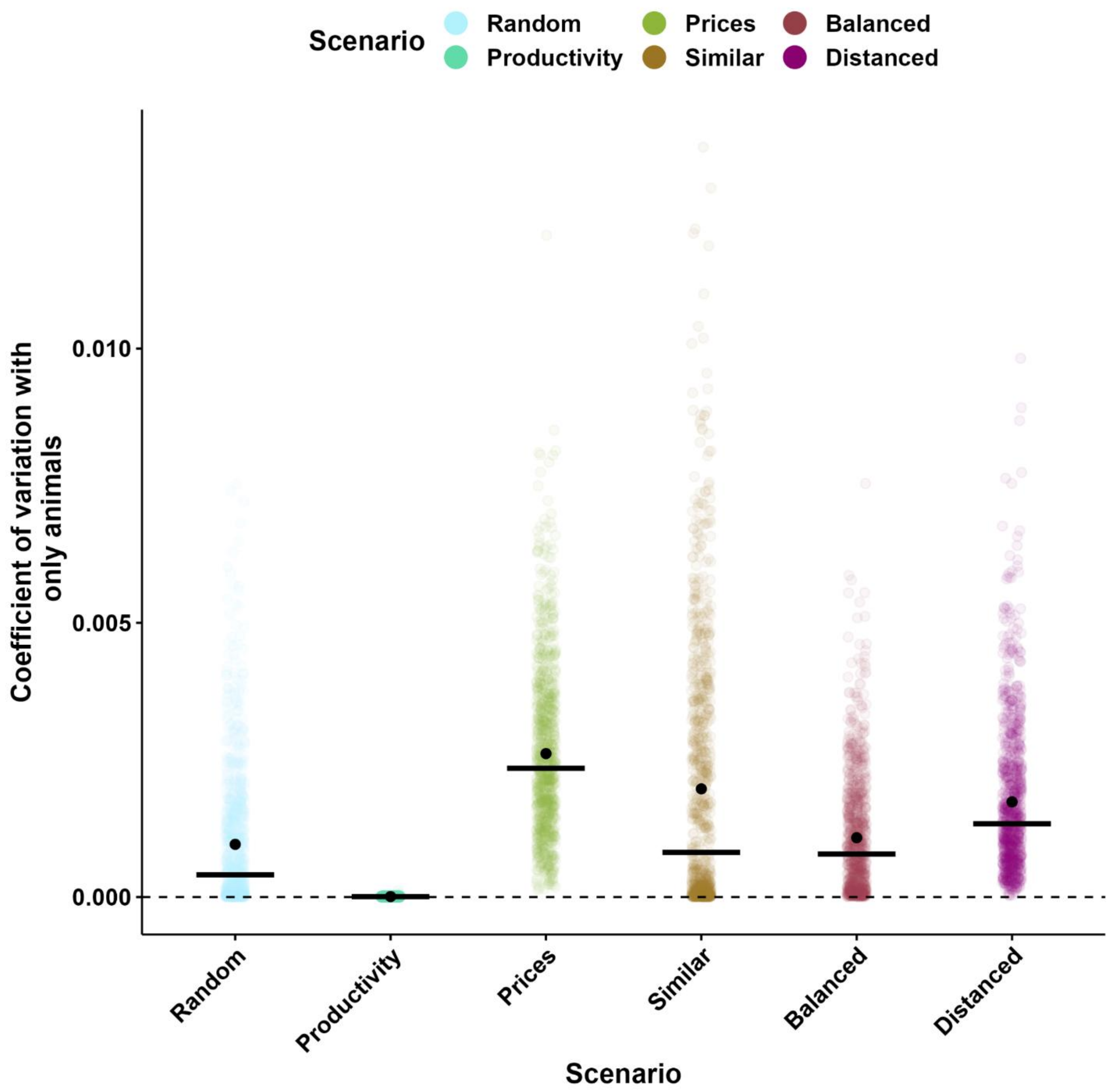

Supplementary Figure S2. We estimate the effect of fisheries on communities using the coefficient of variation of the total species biomass during the 20,000 time steps following the introduction of fisheries. It is defined as the standard deviation of the total biomass of species in food webs divided by its mean value. 800 food webs were simulated for each scenario.

SI 3 Coefficient of variation for animal species

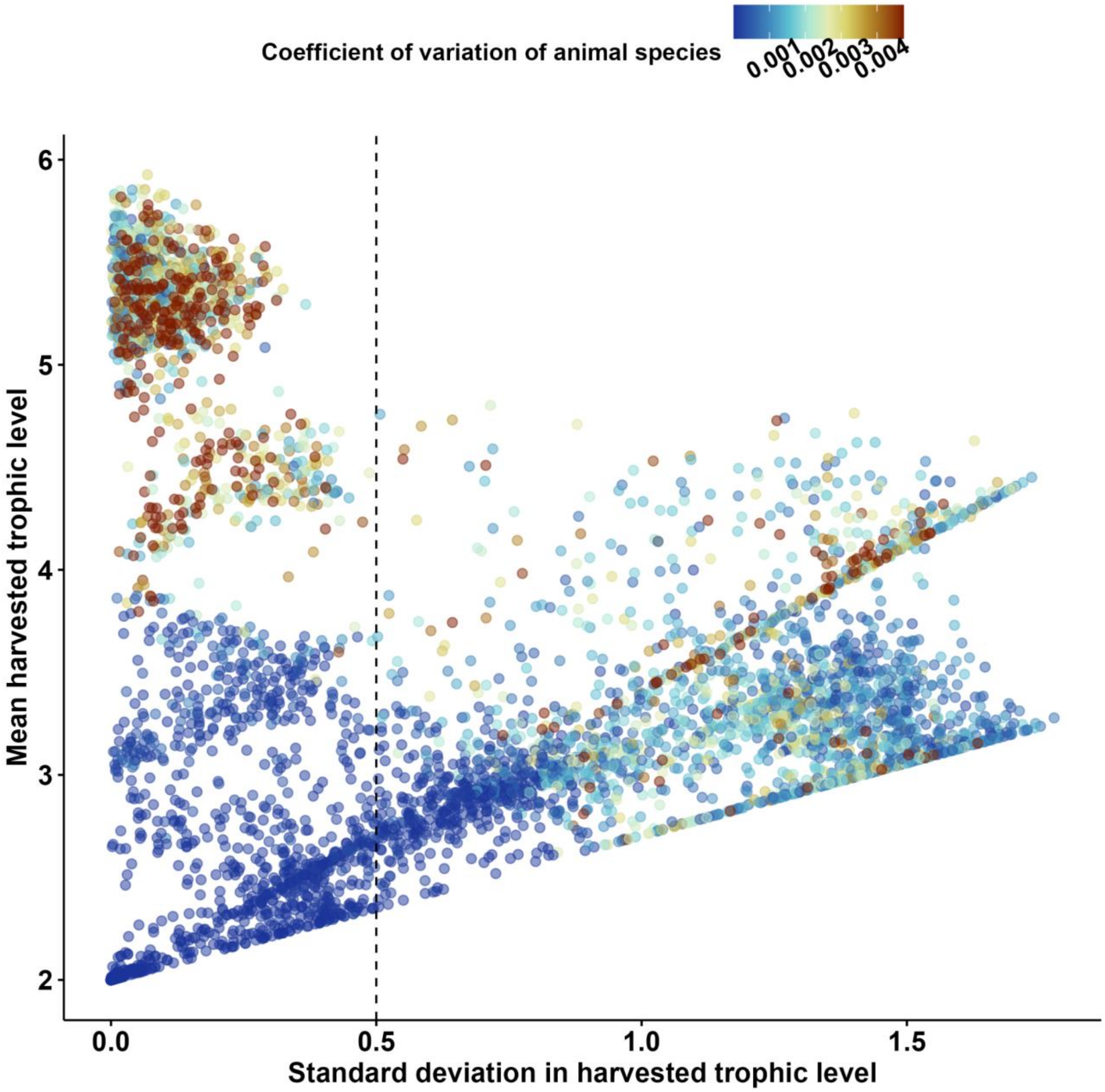

Supplementary Figure S3. We estimate the effect of fisheries on communities using the coefficient of variation of the total species biomass during the 20000 time steps following the introduction of fisheries. It is defined as the standard deviation of the total biomass of species in food webs divided by its mean value. The top and bottom 5% of values were plotted on top as either red (for highest values) or blue (for lowest values). n = 800 food webs.

SI 4 Share of biomass caught compared to combined harvested species biomass

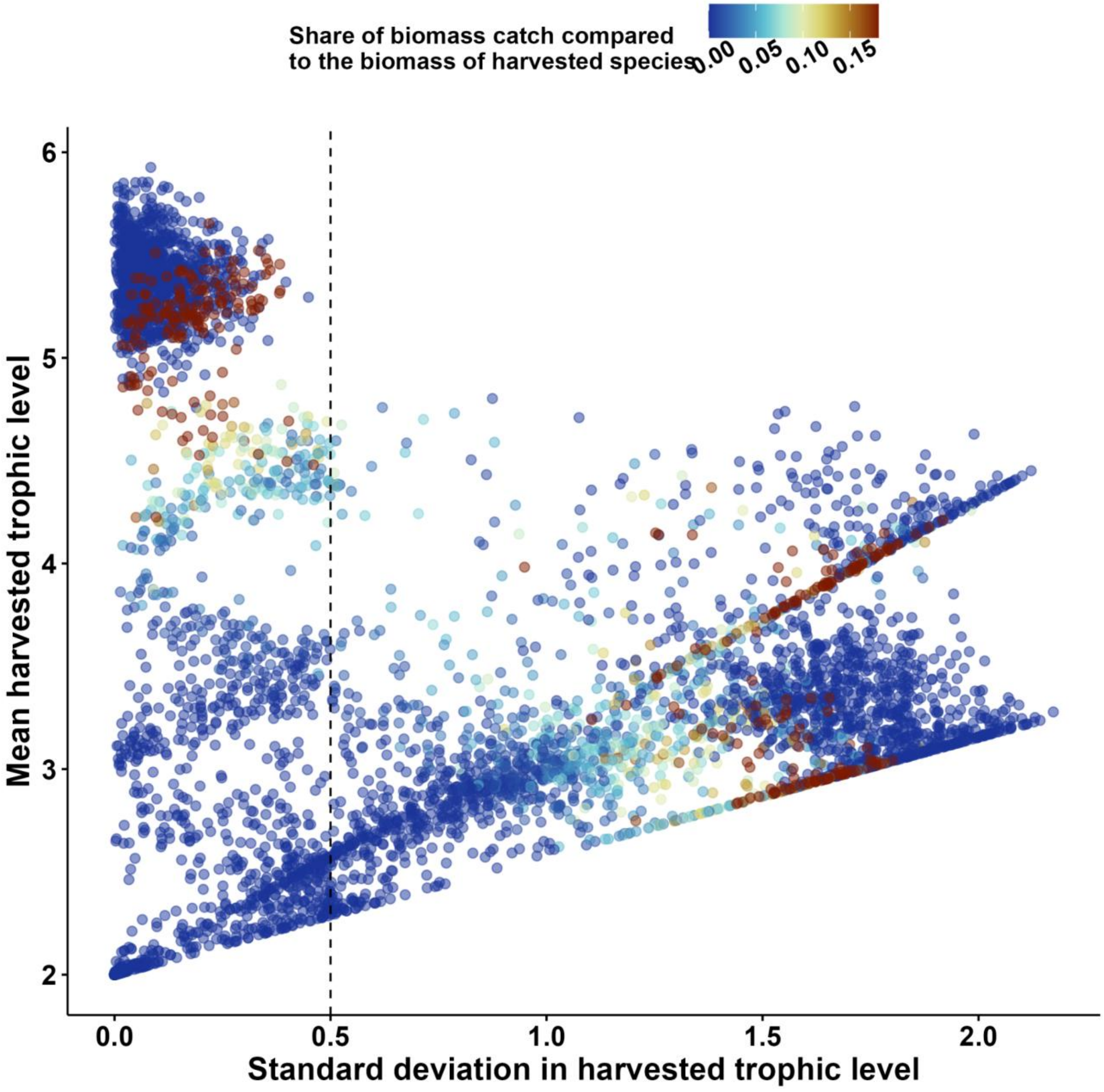

Supplementary Figure S4. The share of the summed biomass caught by all fisheries compared to the summed biomass of the harvested species plotted by the standard deviation in harvested trophic level (x-axis) against the mean harvested trophic level (y-axis). The top and bottom 5% of values were plotted on top as either red (for highest values) or blue (for lowest values). n = 800 food webs.

SI 5 Share of biomass caught compared to food web total biomass

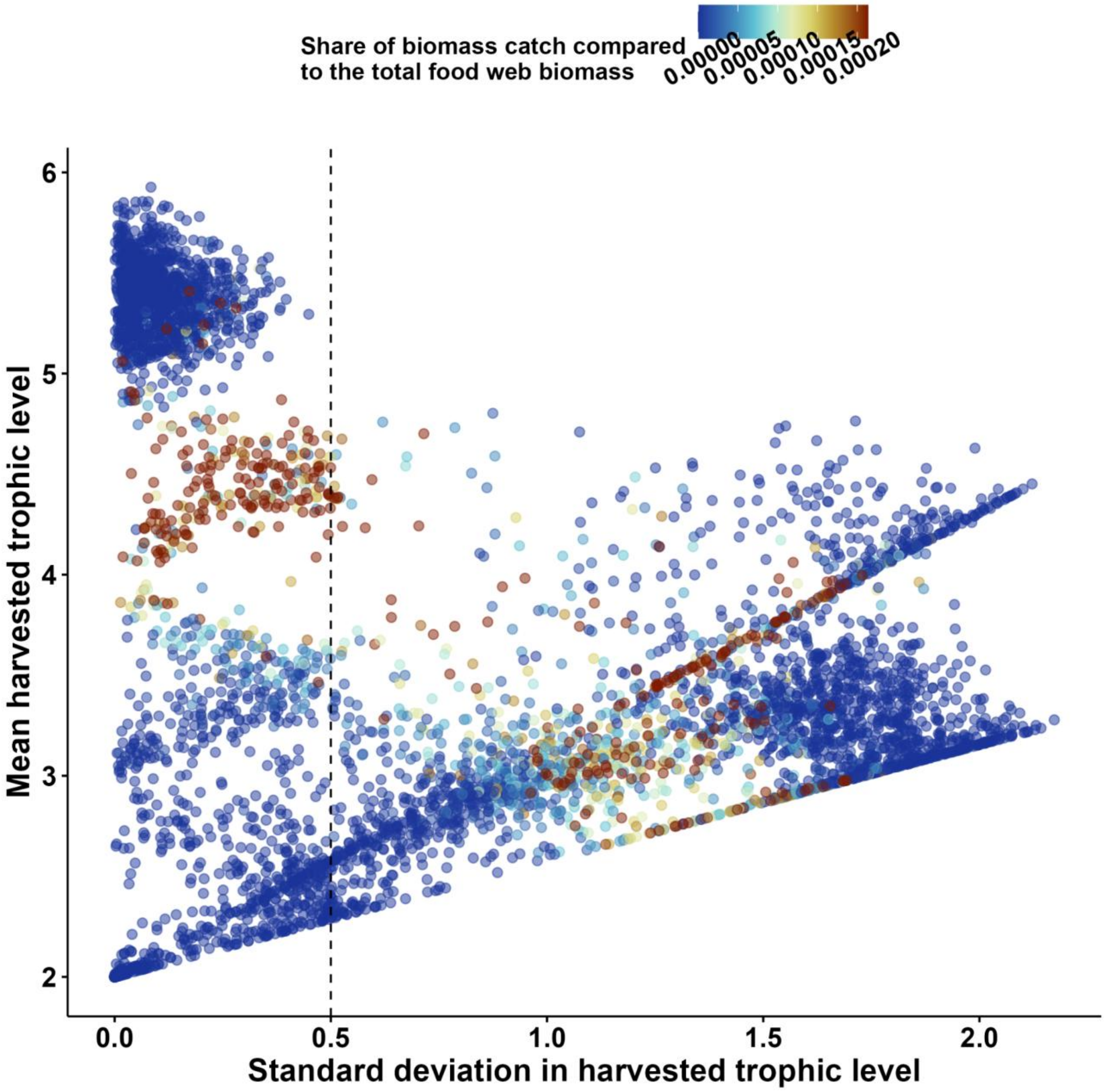

Supplementary Figure S5. The share of the summed biomass caught by all fisheries compared to the biomass of the food web plotted by the standard deviation in harvested trophic level (x-axis) against the mean harvested trophic level (y-axis). The top and bottom 5% of values were plotted on top as either red (for highest values) or blue (for lowest values). N = 800 food webs.

SI 6 Comparing results where trophic levels of invertebrates and vertebrates overlap

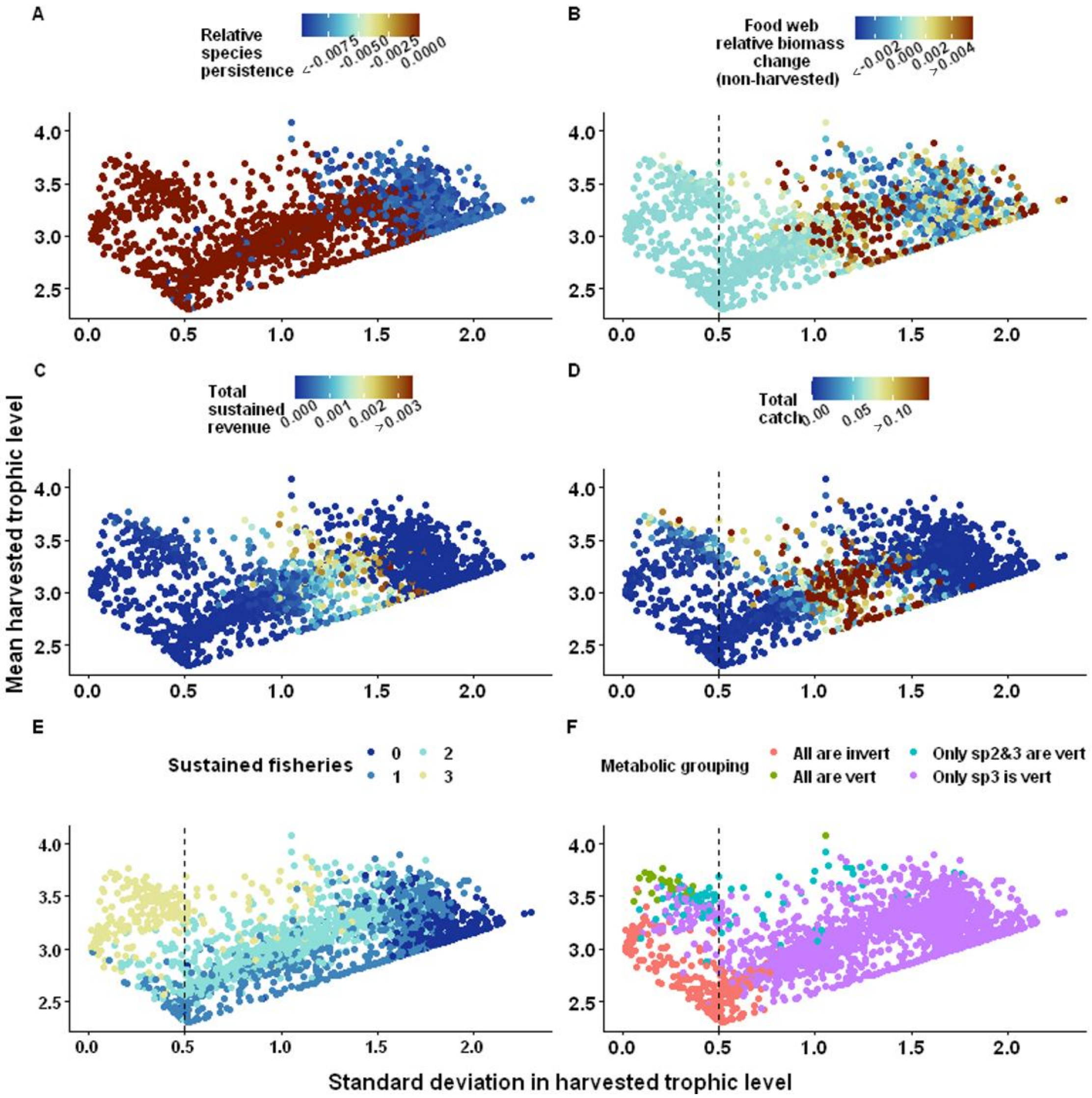

Supplementary Figure S6. Different indicators plotted by the standard deviation in harvested trophic level (x-axis) against the mean harvested trophic level (y-axis). (A) Relative species persistence, defined as the ratio of surviving vs initial species count in a food web at the start of harvest, divided by the initial species count ; (B) Relative food web relative biomass change, excluding harvested species, defined as the summed harvested biomass vs the summed pristine biomass for non-harvested species in a food web, divided by the summed pristine biomass; (C) Total sustained revenue, defined as the sustained revenue sum of all three fisheries; (D) Total fish biomass caught, defined as the sum of biomass caught by all three fisheries; (E) Sustained fisheries, defined as having maintained a density greater than 10-10 throughout the simulation run; and (F) Metabolic groups. Each dot represents one food web. The dashed line at x = 0.5 is given for reference to the upper bound of the scenario Similar. N = 800 food webs.

SI 7 Sensitivity of the number of fisheries

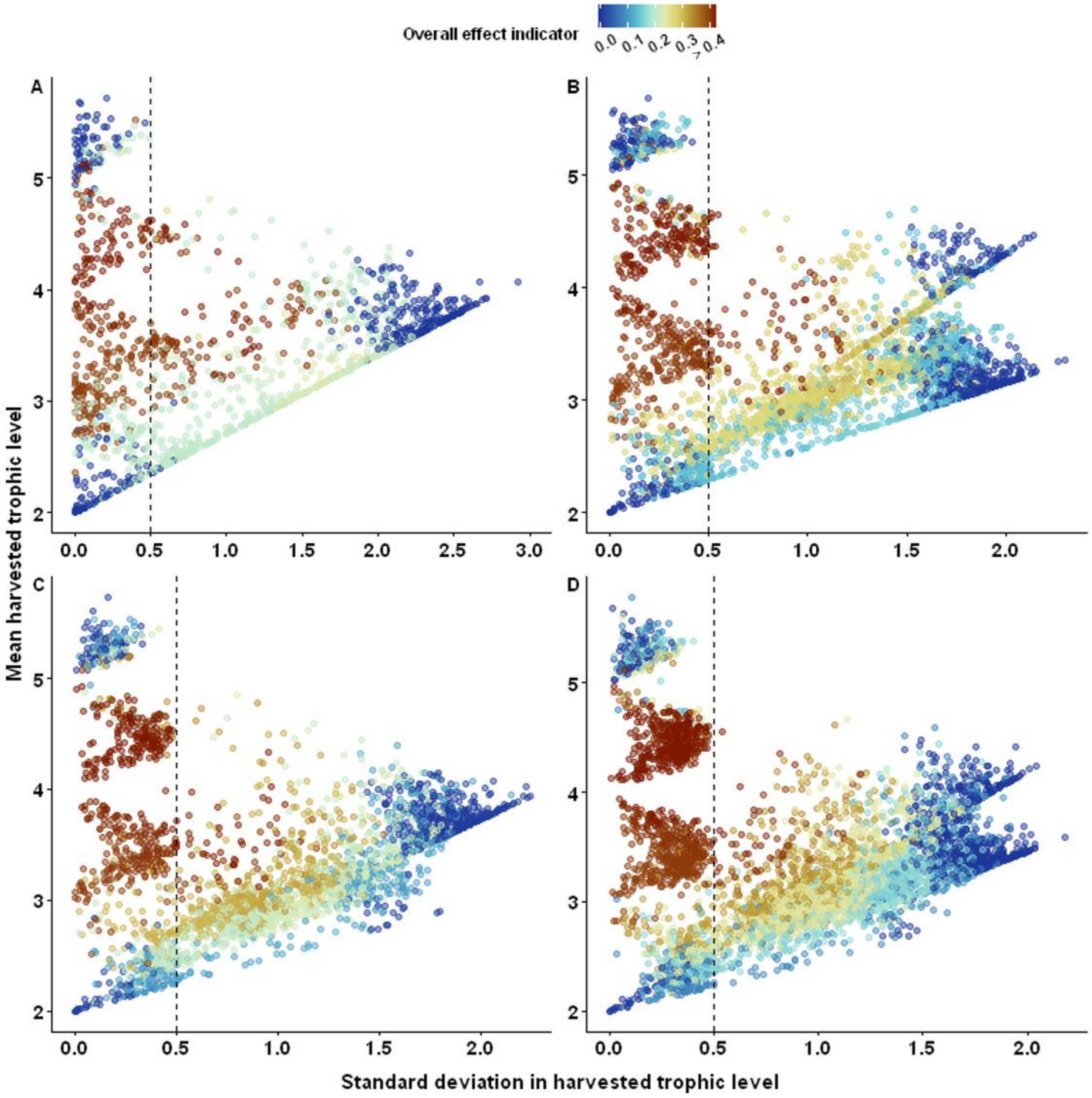

Supplementary Figure S7. Overall effect results for food webs differing in the number of fisheries. (A) Two-fishery system; (B) Three-fishery system; (C) Four-fishery system; and (D) Five-fishery system. The overall indicator is generated from each indicator being scaled to itself (0-1) and the average of the five indicators. Each dot represents one food web. The dashed line at x = 0.5 is given for reference. N = 800 food webs.

SI 8 Sensitivity to price assumption

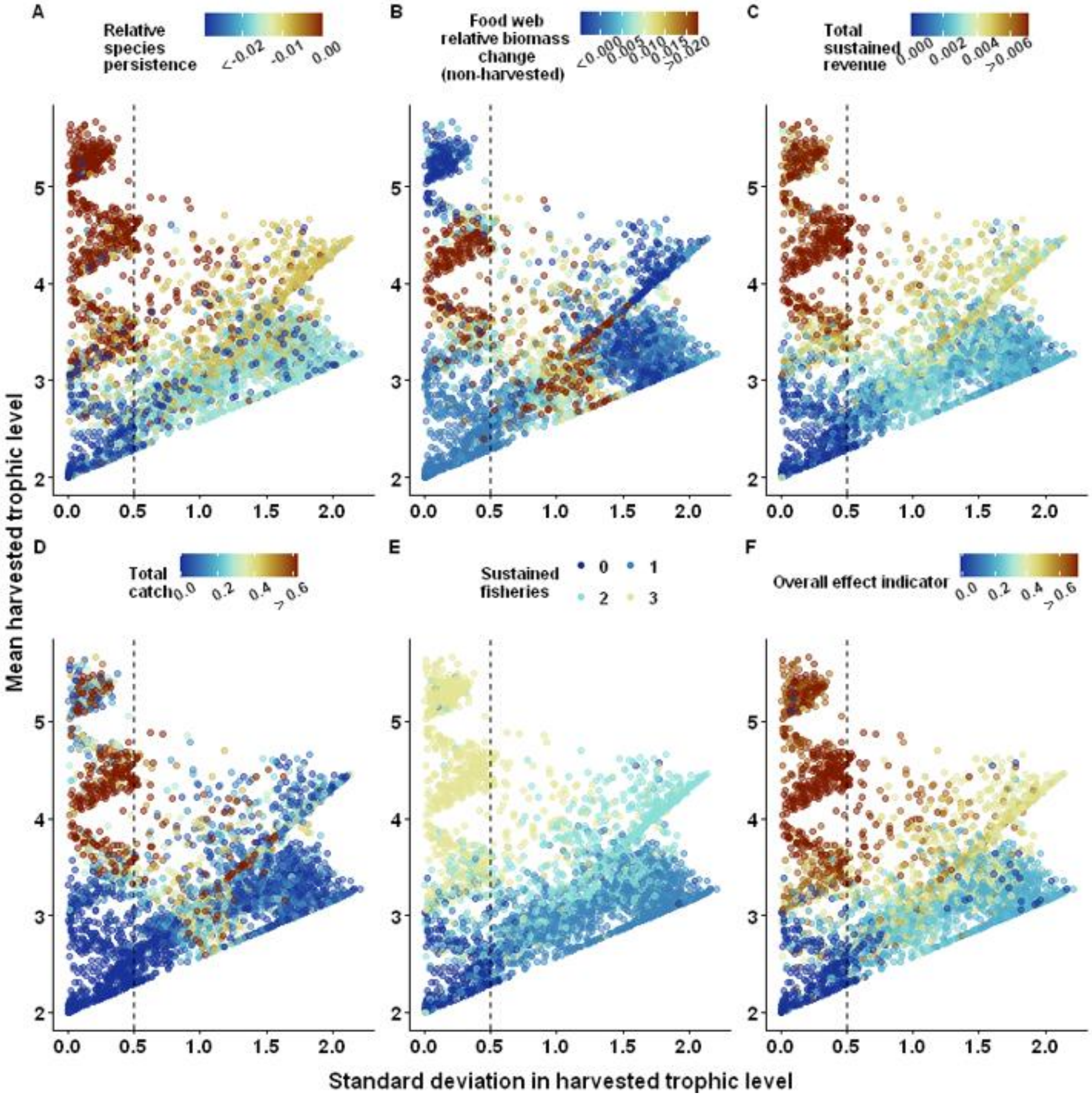

Supplementary Figure S8. Different indicators plotted by the standard deviation in harvested trophic level (x-axis) against the mean harvested trophic level (y-axis) for simulations where base fish price was not adjusted by species body mass. Each indicator (A) Relative species persistence, defined as the ratio of surviving vs initial species count in a food web at the start of harvest; (B) Relative food web biomass change, excluding harvested species, defined as the summed harvested biomass divided by the summed pristine biomass for non-harvested species in a food web; (C) Sustained total revenue, defined as the sustained revenue sum of all three fisheries; (D) Sustained total biomass catch, defined as the sum of biomass caught by all three fisheries; (E) Number of sustained fisheries; and (F) Overall effect indicator, defined as the mean of all self scaled indicators. Each dot represents one food web. The dashed line at x = 0.5 is given for reference. The systematic linear patterns visible are a result of whether the middle species harvested is closer in trophic level to the smallest species (lower edge) or closer to the largest species (upper edge) (See SI 15 for comparison). n = 800 food webs.

SI 9 Persistence after harvesting relative to the initial species richness parameter.

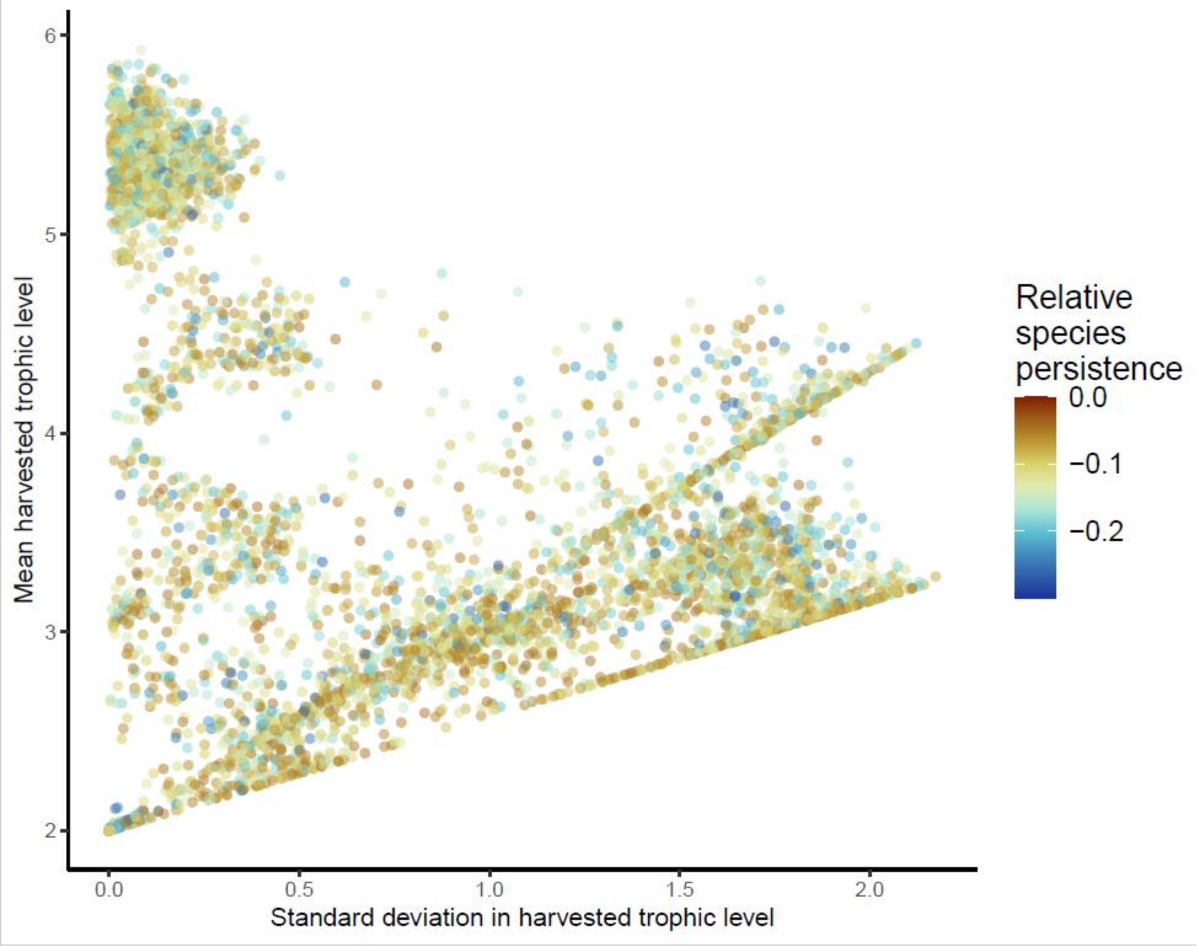

Supplementary Figure S9. Species persistence expressed as species richness at the end of the simulation runs relative to the species richness prior to the pristine run without fisheries. Note the higher variability in relative species persistence, which is caused by the high variability of extinctions during the pristine run irrespective of fishery effects. n = 800 food webs.

SI 10 Feeding motifs

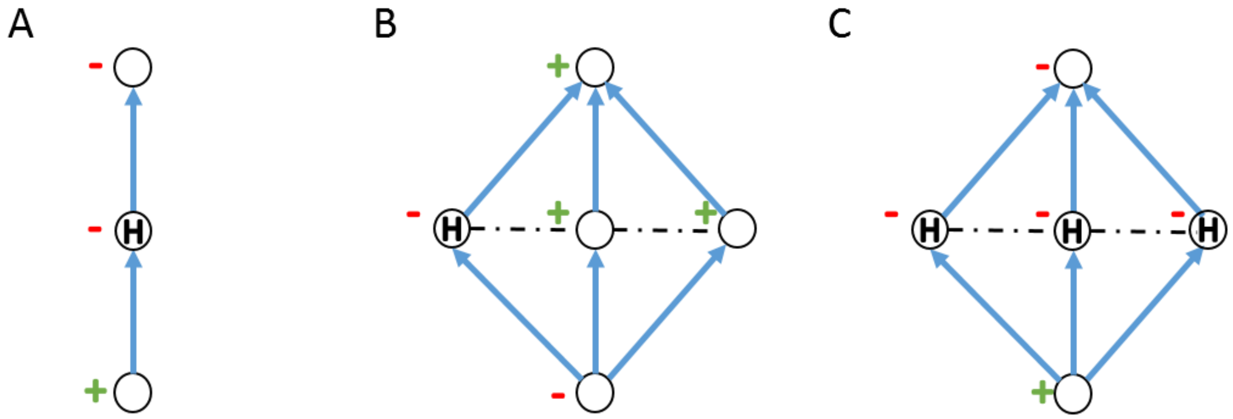

Supplementary Figure S10. Feeding motifs showing species biomass change patterns when a middle species is harvested (H). A - simple food chain; B - Apparent competition effects when competitors are not harvested, C - apparent competition effects when competitors are harvested. The Black dashed line indicates apparent competition.

SI 11 Biomass per rounded trophic level

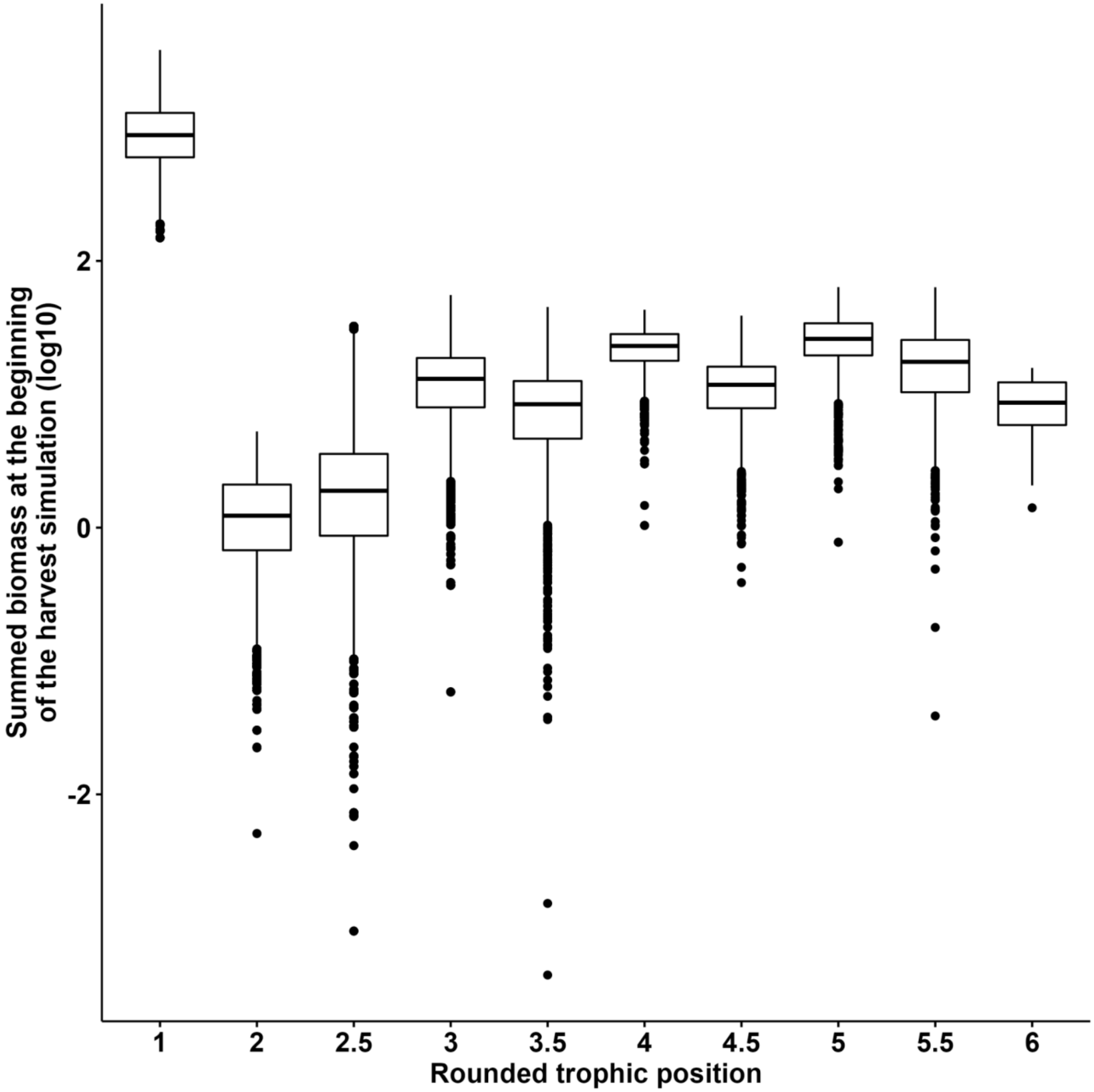

Supplementary Figure S11. The summed species biomass before harvesting starts, grouped by rounded trophic position. n = 800 food webs.

SI 12 Influx per rounded trophic level

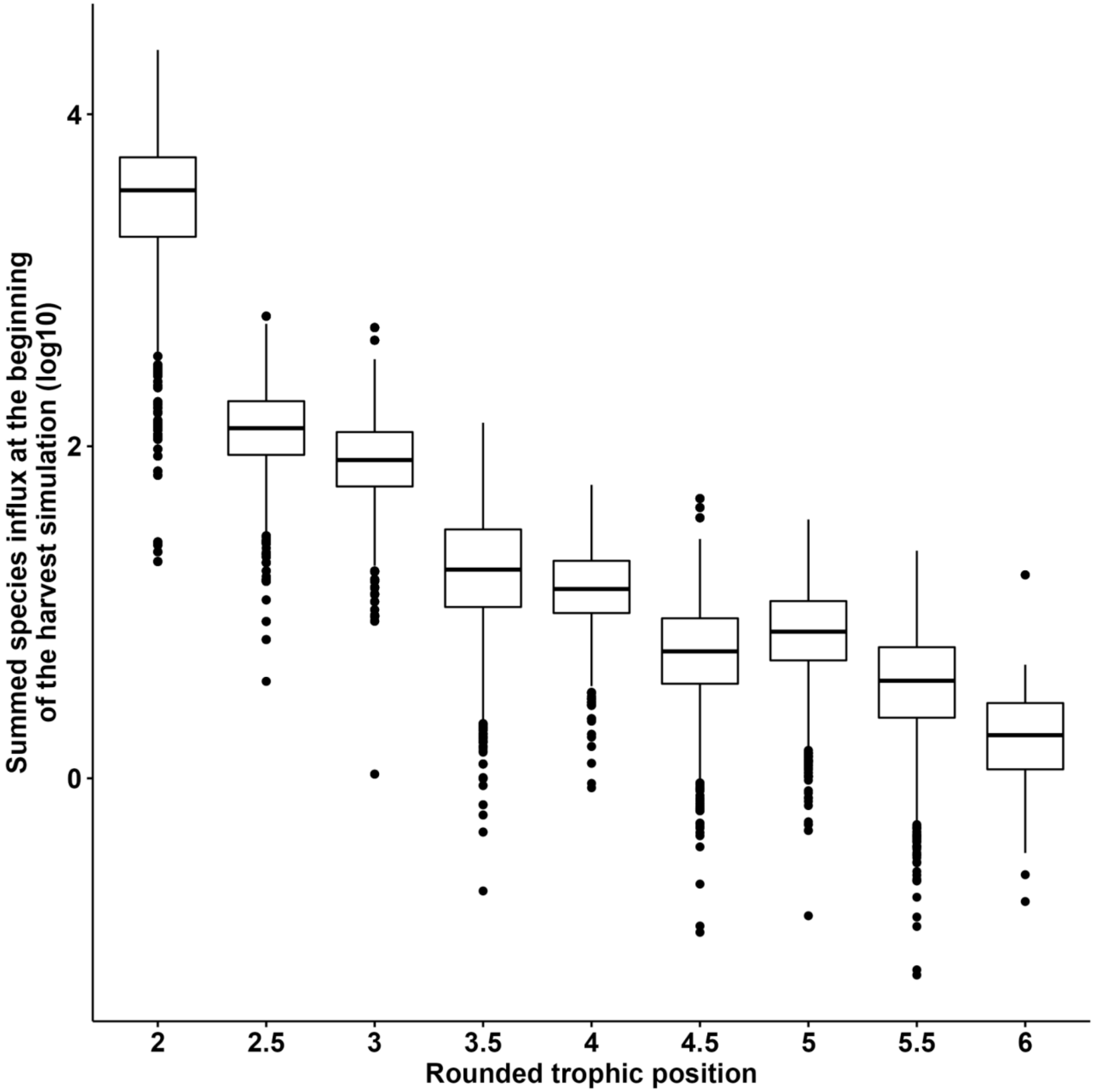

Supplementary Figure S12. The summed species influx, defined as the energy passing through a species node minus the metabolism cost, before harvesting starts, grouped by rounded trophic position. n = 800 food webs.

SI 13 Initial clearance value

9.730694754814426, is based on the intercept of a Type II functional response (**Equation A**) and its inverse (**Equation B**), representing a choice in strategy for a fishery to focus on abundant and less valuable prey (decrease searching costs but also decrease pay off) or focusing on rarer but more valuable prey (increase searching costs but also increase pay off). We expect an adaptive clearance rate to stay within the limits of these two equations. The intercept is the starting clearance value for every vessel.

A. $$clearance_{V_a} = (clearance_{Vmax} - clearance_{Vmin} \frac{)*{B_i}^{\beta V}}{{B_{0V}}^{\beta V} + {B_i}^{\beta V}} + clearance_{Vmin}$$

B. $$clearance_{V_b} = \frac{1}{\frac{\frac{1}{clearance_{Vmax}+clearance_{Vmin}} * {B_i}^{\beta V}}{{B_{0V}}^{\beta V} + {B_i}^{\beta V}} + \frac{1}{clearance_{Vmax}}}$$

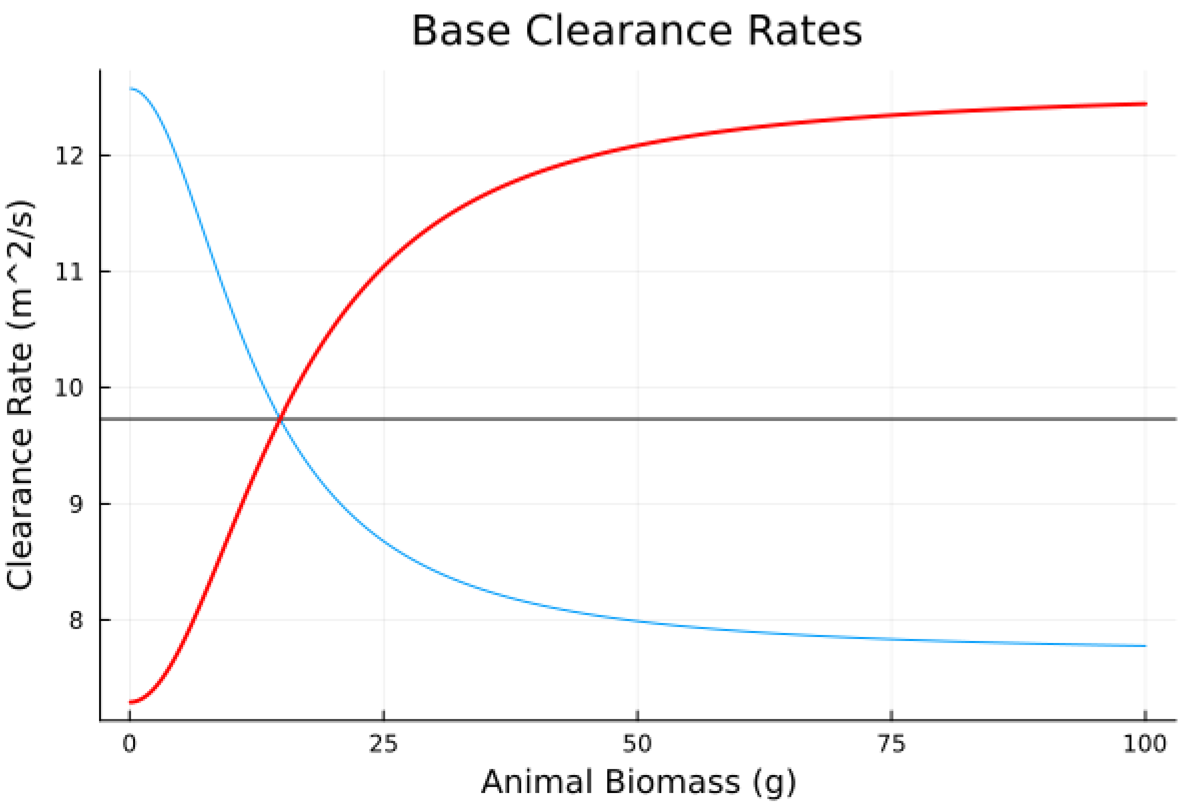

Supplementary Figure S13. An example of initial clearance types and their relation to animal biomass. Equation A - red, Equation B - blue, intercept - black. Taken from Werner et al., 2023.

SI 14 Weighted fish price

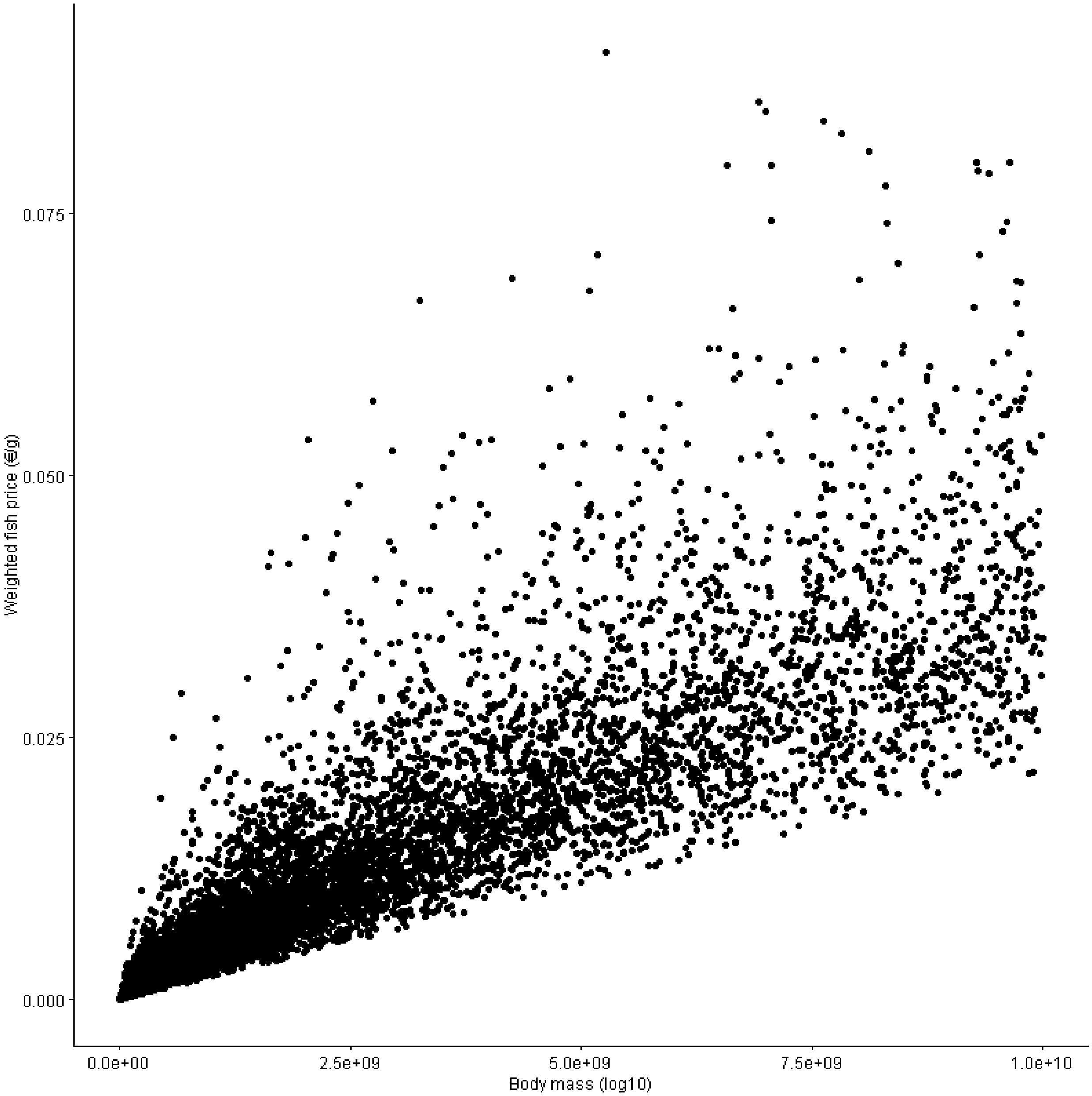

Supplementary Figure S14. Weighted fish price. The weighted fish price (y-axis) is the base price associated with species *i* multiplied by the ratio of body mass of species *i* over the mean consumer body mass in the food web. The body mass of species *i* (x-axis) on a log10 scale.

SI 15 Bottom- or top- focused fishing

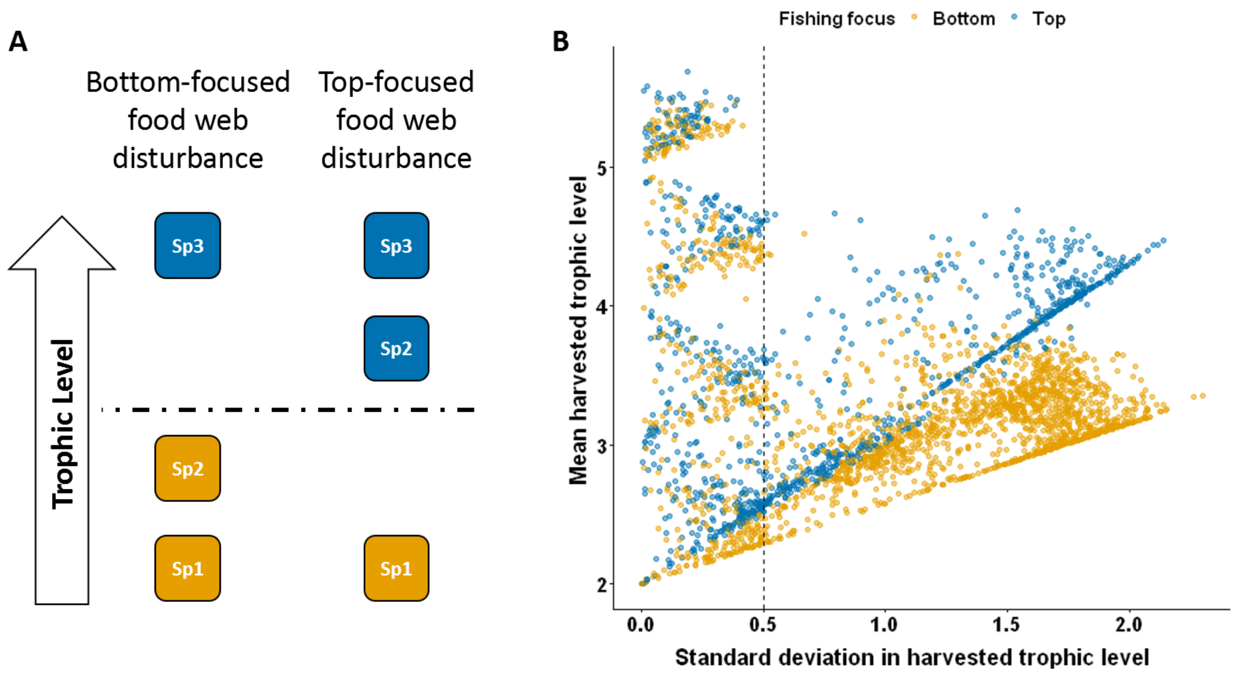

Supplementary Figure S15. Explaining bottom or top fishing focus (A) A food web has a bottom-focused or top-focused disturbance based on whether the middle harvested species (Sp2) is closer in trophic level to the smallest (Sp1) or largest harvested species (Sp3), yellow or blue, respectively; (B) the standard deviation in harvested trophic level (x-axis) against the mean harvested trophic level (y-axis), separated by a bottom or top focused fishing disturbance. Each dot represents one food web. The dashed line at x = 0.5 is given for reference to the scenario Similar.

SI 16 Model variables

Supplementary Table 1 Food web topology

Symbol text	Symbol Julia code	Meaning	Unit	Value	Reference
	P InV V A	Plant Invertebrate Vertebrate Animal	-	-	
	num_nutrient	Number of nutrients	-	2	62
	num_plant	Number of plant species	-	30	62
	num_invertebrate	Number of invertebrate species	-	17 - 67	
	num_vertebrate	Number of vertebrate species	-	8 - 33	
	num_vessel_types	Number of vessel types	-	3	
	foodWeb	Establishes probability of links existing	-	calculated	
B	bioS	Biomass density of plant, animal	$g*m^{-2}$	Initial<br>P and A<br>Uniform dist. (0,10)	
e	exp	Natural logarithm base		2.7182….	-
L	L	Establishes potential links between species	-	Calculated in Eq. 1	
m	bm	Log $_{10}$ body mass	g	Uniform dist.<br>$\mu_P = (10^0, 10^6)$<br>$\mu_{InV} = (10^2, 10^6)$<br>$\mu_V = (10^4, 10^{10})$	62, modified
R_{opt}	Ropt	Optimal consumer-resource body-mass ratio	-	100	62
subscripts $_{I, j, k}$	pred, prey	Consumer relationship between species $_{i,j,k}$	-	-	
γ	γ	Ricker function width	-	2	62

Supplementary Table 2 Ecologic dynamics

Symbol text	Symbol Julia code	Meaning	Unit	Value	Reference
e_k	ae_P , ae_A	Assimilation efficiency		P: 0.545<br>A: 0.906	64
x_i	X	Metabolic demand		Calculated in	
x_i	x_P , x_InV , x_V	Metabolic rate		P: 0.138<br>Inv: 0.314<br>V: 0.88	62<br>62<br>62
a	-	Metabolic rate scaling constant		P: 0.25<br>Inv: 0.15<br>V: 0.11	62<br>62<br>62
r	r	Intrinsic plant species growth rate	$g*s^{-1}$	$r_i = m_i^{-0.25}$	
G	CalcG	Plant species specific growth factor		Calculated in Eq. 3	62
N	bioS[num_nutrient]	Nutrient concentration	$g*m^{-2}$	Initial<br>Uniform dist. (0,10)	
K	K	Half saturation density of nutrient; nutrient uptake efficiency		Uniform dist.<br>0.1-0.2	62
D	D	Global nutrient turnover rate; replenishment rate		0.25	62
S	S	Nutrient supply concentration		Norm. dist.<br>$\mu = 100$, $\sigma = 2$	
v	v	Relative nutrient content in plant species biomass		$v_1 = 1$<br>$v_2 = 0.5$	62
		Extinction threshold		0.000001	62

Supplementary Table 3 Feeding rates

Symbol text	Symbol Julia code	Meaning	Unit	Value	Reference
F_{ij}	FF	Feeding rate or feeding function		Calculated in Eq. 5	
ω	ω	Relative consumption rate<br>(1/# resources for species *i*)		Calculated	
q	q	Hill exponent	-	Norm. dist., μ = 0.5, σ = 0.2	62
c	c	Consumer interference	-	Norm. dist., μ = 0.8, σ = 0.2	62
h	h	Handling time	$g*s^{-1}$	Calculated in Eq. 6	
h_0	h0	Scaling constant		0.4	62
B_0	b0	Capture coefficient; half-saturation density		Herbivores: 3500.0<br>Carnivores: 15.0	62
β	β	Power-law relationship scaling		Herbivores: 0.19<br>Carnivores: 0.42	62
η	η	Scaling constant		Norm. dist.<br>ηprey : μ = -0.48, σ = 0.03<br>ηpred: μ = -0.66, σ = 0.02	62
b_{ij}	b	Resource specific capture coefficient		Calculated in Eq. 7a,b	

Supplementary Table 4 Humans as a food web node

Symbol text	Symbol Julia code	Meaning	Unit	Value	Reference/rationale
active fishing	active_fishing	Fishing hours spent actively fishing (fraction)	-	0.9	Assume large scale fisheries being active 90% of time.
B_{0V}	b0V	Vessel half saturation density	$g*m^{-2}$	16.0	Selected for allowing regular coexistence between fisheries and harvested species
$catch_i$		Total biomass caught	$g*s^{-1}$	Calculated in Eq. 8	
$catch\_max_i$	catch_max	Maximum biomass a vessel can catch	$g*s^{-1}$	200.0	S1, increased from an order of magnitude to simulate large scale fisheries
$clearance_i$	V_clearance	the surface area a vessel can clear of fish	m^2*s^{-1}	Initial 9.730694754814426	SI 14
$clearance_{Vmax}$	V_clearance_max	Maximum vessel clearance rate	m^2*s^{-1}	fish_hour* fish_active* fish_day_max* gear	
$clearance_{Vmin}$	V_clearance_min	Minimum vessel clearance rate	m^2*s^{-1}	fish_hour* fish_active* fish_day_min* gear	
$cost_i$	cost	Cost of one vessel	$€*s^{-1}$	Calculated in Eq. 9	
$fishing\ days_{max}$	fish_day_max	Maximum days per year spent fishing (fraction)	-	0.69	S2
gear		Vessel equipment: net size and vessel speed	m^2*s^{-1}	V_speed * fish_net	
$fishing\ days_{min}$	fish_day_min	Minimum days per year spent fishing (fraction)	-	0.40	S2
fishing hours	fish_hour	Hours per day spent fishing (fraction)	-	0.9	Free parameter
$maintenance_i$	maintenance	Cost of maintaining a vessel ready to leave port	$€*s^{-1}$	0.06	Selected for allowing regular coexistence between fisheries and harvested species
Net_{width}	fish_net	Fishing net width	m	15.0	9
p_{Base}	γ_fish	Fish price coefficient	$€*g^{-1}$	0.00129	S4
PED	elasticity	How sensitive the market price of fish is to a change in fish supply	-	-1.15	S3
p_i	fish_price	Price the market will buy fish at	$€*g^{-1}$	Calculated in Eq. 10	

$revenue_i$	revenue	Selling all caught fish	€*s^{-1}	Calculated in Eq. 12	
scaling	cost_scale	How costs scale in relation to the clearance rate	€*m^{-2}	0.07	Selected for allowing regular coexistence between fisheries and harvested species
V_i	bioS[(num_nutrient+num_plant+num_animal+num_vessel_types)]	Area covered by the fleet	1/*m^{-2}	Initial Randomly drawn [0.0001,0.001]	S5
V_{speed}	V_speed	Average vessel speed	m/s	1.5	9
β_V	β_V	Scaling exponent for clearance	-	2.0	Selected for allowing regular coexistence between fisheries and harvested species
μ_c	μ_c	How quickly clearance changes based on profit	m^2*$€^{-1}$*s^{-1}	μ_v * 30	Derived from $\mu_v$to assume faster adaptation of foraging in comparison to number of vessels
μ_v	μ_v	How quickly number of vessels changes based on profit	m^2*$€^{-1}$*s^{-1}	0.01	26
π_i	Profit	Positive or negative profit based on whether demand price covers cost of fleet	€*s^{-1}	Calculated in Eq. 13	

Active Fishing

The active fishing parameter reflects the ability of fishing vessels to locate and engage in fishing activities efficiently. For large-scale fisheries, it was set to 0.9, under the assumption that large vessels, with advanced equipment and large crews, can begin fishing rapidly and sustain operations effectively.

Catch_max

The maximum catch rate (•Catchmax) was derived from Ayunda et al. (2018), which identified a baseline of 23 g/s for small-scale fisheries. For large-scale fisheries, this parameter was adjusted upwards to 200 g/s to account for their higher capacity and efficiency.

Clearance

The clearance rate, which influences the interaction strength between fishing fleet and prey, was initially defined using data from SI14. It was based on the intercept of a Type II functional response curve and its inverse.

Fishing Hours

For large fisheries, fishing hours were set at 0.9, referencing data from the International Labour Organization (ILO, 2004).

Maintenance

The maintenance cost parameter reflects the operational expenses of fishing vessels. For large-scale fisheries, this value was set to 0.06 €/s, accounting for the high costs associated with advanced equipment and large crews (based on FAO, 2001).

Base Price (P_base)

The base price of fish was determined using the mean value of all species of fresh fish and mollusks caught in Indonesia in 2015, which was 1.29 US$/kg. Given the approximate parity between the Euro and US dollar, this was converted to 0.0129 €/g. This represents the price for fish resource that is reaching the market. Given the high percentage of loss of fish biomass after the catch and strong spatial and temporal fluctuations in prices, an additional adjustment was made, resulting in a final value of 0.00129 €/g.

Mortality Rate (μ_v)

The mortality rate of fishing vessels (•μ_v) was informed by research from Glaum et al. (2020). This parameter reflects the likelihood of vessels exiting the fishery, either through economic failure or operational constraints.

Supplementary References:

[S1] AYUNDA, Nisa, SAPOTA, Mariusz R., et PAWELEC, Anna. The impact of small-scale fisheries activities toward fisheries sustainability in Indonesia. *Interdisciplinary Approaches for Sustainable Development Goals: Economic Growth, Social Inclusion and Environmental Protection*, 2018, p. 147-167.

[S2] GUIET, Jérôme, GALBRAITH, Eric, KROODSMA, David, *et al.* Seasonal variability in global industrial fishing effort. *PLoS One*, 2019, vol. 14, no 5, p. e0216819.

[S3] COSTELLO, Christopher, OVANDO, Daniel, CLAVELLE, Tyler, *et al.* Global fishery prospects under contrasting management regimes. *Proceedings of the national academy of sciences*, 2016, vol. 113, no 18, p. 5125-5129.

[S4] SEAFDEC, GEF. *"Strategies for Trawl Fisheries Bycatch Management" GCP/RAS/269/GFF REBYC-II CTI Indonesia, Regular Meeting for National Working Group (NWG) REBYC-II CTI 10 September 2015*. SEAFDEC, 2015.

[S5] WERNER, Alexandra S., Hirt, Myriam, Ryser, Remo, *et al.* Maintaining ecological stability for the sustainable economic yield of multispecies fisheries in complex food webs. 2023.
